# Machine Learning-Based Identification of Mating Type and Metalaxyl Response in *Phytophthora infestans* Using SSR Markers

**DOI:** 10.3390/microorganisms12050982

**Published:** 2024-05-14

**Authors:** Collins A. Agho, Jadwiga Śliwka, Helina Nassar, Ülo Niinemets, Eve Runno-Paurson

**Affiliations:** 1 Institute of Agricultural and Environmental Sciences, Estonian University of Life Sciences, Kreutzwaldi 1, 51006 Tartu, Estonia; 2 Plant Breeding and Acclimatization Institute—National Research Institute in Radzików, Department of Potato Genetics and Parental Lines, Platanowa Str. 19, 05-831 Młochów, Poland; 3 Estonian Academy of Sciences, Kohtu 6, 10130 Tallinn, Estonia

**Keywords:** *Phytophthora infestans*, molecular markers, mating type, fungicide resistance, machine learning

## Abstract

*Phytophthora infestans* is the causal agent of late blight in potato. The occurrence of *P. infestans* with both A1 and A2 mating types in the field may result in sexual reproduction and the generation of recombinant strains. Such strains with new combinations of traits can be highly aggressive, resistant to fungicides, and can make the disease difficult to control in the field. Metalaxyl-resistant isolates are now more prevalent in potato fields. Understanding the genetic structure and rapid identification of mating types and metalaxyl response of *P. infestans* in the field is a prerequisite for effective late blight disease monitoring and management. Molecular and phenotypic assays involving molecular and phenotypic markers such as mating types and metalaxyl response are typically conducted separately in the studies of the genotypic and phenotypic diversity of *P. infestans*. As a result, there is a pressing need to reduce the experimental workload and more efficiently assess the aggressiveness of different strains. We think that employing genetic markers to not only estimate genotypic diversity but also to identify the mating type and fungicide response using machine learning techniques can guide and speed up the decision-making process in late blight disease management, especially when the mating type and metalaxyl resistance data are not available. This technique can also be applied to determine these phenotypic traits for dead isolates. In this study, over 600 *P. infestans* isolates from different populations—Estonia, Pskov region, and Poland—were classified for mating types and metalaxyl response using machine learning techniques based on simple sequence repeat (SSR) markers. For both traits, random forest and the support vector machine demonstrated good accuracy of over 70%, compared to the decision tree and artificial neural network models whose accuracy was lower. There were also associations (*p* < 0.05) between the traits and some of the alleles detected, but machine learning prediction techniques based on multilocus SSR genotypes offered better prediction accuracy.

## 1. Introduction

Potatoes (*Solanum tuberosum*, Solanaceae) are one of the most significant vegetables worldwide [1]. Late blight *Phytophthora infestans* (*P. infestans*) is a pathogen that affects field-grown potatoes and tomatoes all over the world and has historically been common in many countries [2,3,4,5,6,7,8]. Throughout the growing season, *P. infestans* can cause water-soaked lesions on the plant leaves, stems, and fruits, with an overall effect of reducing yield quality and quantity [9,10]. It is the most devastating disease to this crop, posing a threat to global food security [10,11,12].

A better understanding of the population dynamics in *P. infestans* has been made possible by diversity studies that take into account both phenotypic and genotypic markers [6,13,14,15,16]. Mating type, virulence spectrum, and metalaxyl/mefenoxam resistance are frequent phenotypic markers [17,18]. Molecular markers such as simple sequence repeat (SSR) markers, amplified fragment length polymorphism (AFLP), and random fragment length polymorphism (RFLP) are commonly employed in *P. infestans* studies due to their high repeatability [6,17,19]. *P. infestans* has two mating types, A1 and A2, and it can reproduce both asexually and sexually. Sexual reproduction can occur when both mating types are present [20]. With the exception of Mexico, which was thought to be the center of origin where the A1 and A2 mating types co-exist, only the A1 mating type isolates were known to occur until the early 1980s [21]. However, as the A2 mating type spread throughout Europe and other parts of the world, sexual reproduction became more common, increasing the genetic diversity and adaptability of the pathogen, resulting in the formation of oospores that can survive for a long time in the soil, even during the winter, and can infect other plants the following season [21,22,23,24,25]. 

Since the 1970s, phenylamide fungicides (initially, the racemic mixture of metalaxyl was used prior to the introduction of its biologically active enantiomer R-metalaxyl (also known as mefenoxam)) have made substantial contributions to the efficient management of *P. infestans* [6,12,18,26,27]. However, fungicide resistance to late blight is a problem that is getting worse due to the introduction of novel strains that are tolerant or resistant to metalaxyl. As early as the 1980s, reports of *P. infestans* isolates in Europe that are resistant to metalaxyl were published [28,29]. The resistance can result from inactivation, altered transport, altered metabolism of the fungicide or modification of the fungicide target as a result of pathogen mutations [30,31,32]. Frequent use of fungicides can create fungicide selection pressure, which can lead to a shift from sensitivity to resistance over time [33,34]. Though the molecular mechanism of fungicide resistance is still not fully understood [12,26], some DNA markers linked to this trait have been suggested [26,35,36]. Ref. [37] reported a complicated inheritance pattern involving both major and minor loci for metalaxyl insensitivity in *P. infestans*, indicating that metalaxyl resistance is controlled by multiple loci. 

The conventional method for determining *P. infestans* response to metalaxyl relies on a standard microbiological assay, in which control and fungicide-amended media are inoculated with the *P. infestans* and the growth of the colonies is measured and compared after 7–14 days [15,38]. To determine the mating types, matching an unknown isolate with tester isolates on an agar plate and watching for oospore formation is used [39]. Despite being straightforward, this method necessitates the isolation and use of live *P. infestans* strains [40]. This method can be time-consuming, requiring 10 to 14 days to ascertain oospore formation, and it prevents the determination of mating-type information on dead biological materials [40]. 

Molecular and phenotypic assays involving molecular and phenotypic markers such as mating types and metalaxyl response are typically conducted separately in the studies of the genotypic and phenotypic diversity of *P. infestans*. It is critical to have a faster methodology that includes swift phenotyping for fungicide response and mating type, while also saving cost when assessing *P. infestans* populations for diversity studies in the event of a potential outbreak or study of the pathogen dynamics, considering its devastating nature [41]. We argue that molecular markers can be utilized to assess not only genotypic diversity but the same marker profile can also be used to determine the mating type and fungicide response using machine learning techniques. This will allow for a quicker analysis of genotypic and phenotypic changes in the population of *P. infestans*, and the technology could lead to optimum management strategies [12], especially when mating type and metalaxyl resistance data are not available, such as in the case of dead isolates. Molecular markers, including SSR markers, have been used for pathogen differentiation, genomic prediction of resistance to pathogens, and characterization of various organisms [42,43,44,45,46]. In previous studies, the use of SSR, inter-simple sequence repeat (ISSR), and sequence-related amplified polymorphism (SRAP) markers gave a good classification for mefenoxam susceptibility and pathotype classification in *Pseudoperonospora cubensis* (*P. cubensis*) [43]. Machine learning techniques have been helpful, with reasonable accuracy, in reducing the workload for modeling and prediction, and molecular and genetic characterization in microbiological and agricultural applications [43,46,47]. For example, notable accuracy was achieved with a particle swarm optimization (PSO) trained artificial neural network (ANN) in the identification of fungicide resistance levels in *P. cubensis* [43]. For cultivar characterization in potato, ANN was also relatively accurate [47]. In another study, the support vector machine (SVM) and Naive Bayes machine learning models were employed to accurately identify cultivars of olive (*Olea europaea*) trees based on Random Amplified Polymorphic DNA (RAPD) and ISSR genetic markers [48].

The application of machine learning techniques to empirical datasets generated from population genetics experiments seems to have received less attention than it may have deserved, given its practical effectiveness [46]. Sexual reproduction is crucial in the biology and epidemiology of *P. infestans*. *P. infestans* populations with A1 and A2 mating types have the capacity to undergo sexual reproduction. This can enhance their longevity and evolution of strains with higher virulence and resistance to common fungicides such as metalaxyl [49]. Therefore, a quick genetic test to assess the prevalence of A1 and A2 mating types, and fungicide-resistant isolates in field populations of *P. infestans* can reduce the workload associated with microbiological assays, and save time and cost, especially in disease monitoring and management. DNA markers such as AFLP markers have been used to identify mating types [49], and metalaxyl resistance [35]. *P. infestans* fungicide resistance has also been determined using Fourier Transform Infra-Red spectroscopy (FTIR) [12]. However, there are no machine learning approaches for mating type and metalaxyl response identification using SSR markers. Our objective was to assess the general efficiency of SSR markers with machine learning approaches and evaluate the efficiency of different machine learning methods in classifying mating type and fungicide response in diverse populations of *P. infestans*.

## 2. Materials and Methods

SSR markers were used to characterize *P. infestans* isolates from Estonia, Pskov region (Russia) (published previously by [6,27], and Poland (published previously by [50,51]. These isolates were genotyped based on the following 12 SSR markers: D13, G11, Pi04, Pi4B, Pi63, Pi70, SSR2, Pi02, SSR4, SSR6, SSR8, and SSR11. The isolates were also phenotyped for mating type (A1 and A2 mating types) and metalaxyl response (resistance, intermediate, and sensitive). For mating type, a total of 850 isolates consisting of 141 isolates from the Pskov region, 111 isolates from Estonia, and 598 isolates from Poland were used. In total, there were 566 A1 and 284 A2 mating types. For metalaxyl response, a total of 823 isolates consisting of 120 isolates from the Pskov region, 111 isolates from Estonia, and 592 isolates from Poland were used. In total, there were 122 isolates of intermediate response, 126 that were resistant, and 575 that were sensitive to metalaxyl. 

## 3. Data Preparation

**Figure 1:**
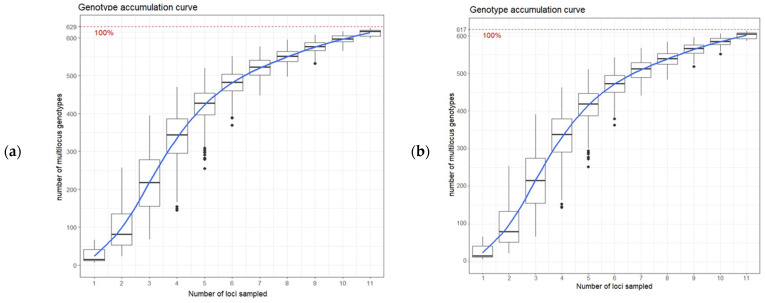
Genotype accumulation curve for the *P. infestans* population for mating type dataset (**a**), and metalaxyl dataset (**b**) over 12 loci. The horizontal axis represents the number of loci randomly sampled without replacement up to (n − 1) loci, while the vertical axis shows the number of multilocus genotypes observed. The red dashed line represents 100% of the total observed multilocus genotypes.

The data were clone-corrected for mating type and metalaxyl resistance using the package *poppr* [52,53] in R (ver. 4.2.0, R Core Team, Vienna, Austria, 2022 [54]). The R package *poppr* was also used to plot a genotype accumulation curve and assess the minimum number of loci that can sufficiently differentiate between different genotypes in the *P. infestans* population [53]. The curve shows a greatly decreased variance over 12 SSR markers and correctly identifies 100% of the multilocus genotypes, indicating its sufficiency for complete genotyping (Figure 1a,b). After correction to remove duplicate genotypes, 629 isolates for mating type and 617 isolates for metalaxyl response remained. 

In total, there were 99 isolates with intermediate response, 105 isolates were resistant, and 413 isolates were sensitive to metalaxyl, respectively. For mating types, 403 isolates were of the A1 mating type and 226 isolates were of the A2 mating type. For predictions, two comparisons were made where one involves using all the alleles for classification and the other uses only common alleles among the populations (Estonia, Pskov region, and Poland). There were 105 alleles in total for both traits (Table S1). Alleles that were specific to each of the populations were removed from the dataset, leaving only the common alleles among the populations. In total, there were 32 common alleles among Estonia, Pskov region, and Poland *P. infestans* isolates for mating type and 31 common alleles for metalaxyl response. Alleles were recoded as 0 for the absence of the allele, 1 for the presence of one copy of the allele, or 2 for the presence of two copies of the allele per locus.

## 4. Machine Learning Methods

The artificial neural network (ANN), support vector machine (SVM), random forest, and decision tree classifier were used as model approaches in this study. Multilayer perceptron (MLP) is a straightforward, deep, feed-forward artificial neural network with three layers (the input, hidden, and output layers), and the neurons of a layer are fully connected to the neurons of the next layers [55]. Each neuron determines its output signal by applying an activation function to its net input, which is the sum of weighted input signals [56]. The artificial neural network (ANN) is an extension of the MLP with more than one hidden layer [57]. ANNs, without having to explicitly explain the complex relationships in the feature space, as required by classical statistical techniques, can tackle non-linear complex classification challenges [58]. This study uses an ANN consisting of the input layer that holds data entering the neural network, three hidden layers that perform nonlinear transformations of the inputs entered into the network, and finally, an output layer that produces the output variables [59,60]. The support vector machine approach is based on statistical learning theory and provides a powerful tool to address the problem of classification [56]. Within two classes of data, SVM determines the hyperplane with the increased margin [57]. Its improved generalization performance is based on the Structural Risk Minimization (SRM) principle [56]. On the other hand, random forest is simple, yet effective in classification and regression, and uses multiple decision trees during the classification process to obtain more accurate results [61]. Random forest creates a model to explain the classification by the predictor variables (traits) using a training subset of the original dataset (in this case, isolates) [61]. A decision tree is a classification method that uses a set of tests that are established at each branch (or node) in the tree to recursively divide a dataset into smaller groups [62]. Since it is strictly nonparametric, no assumptions about the distributions of the input data are necessary [62], and it is a dominant and acceptable method for classification and prediction [57]. For both traits (mating type and metalaxyl response), 70% of the dataset was used as the training set, while the remaining 30% was used as the testing set. The random forest, decision tree classifier, support vector machine, and ANN were implemented using Python modules within the Jupyter Notebook interface. For the ANN, three hidden layers were used with a depth of four (three hidden layers, and one output layer, excluding the input layer) [60]. The activation function sigmoid was used as the output layer for the mating type (binary outcome), while for the metalaxyl response (more than two categories), the activation function softmax was used as the output layer [60]. Accuracy of performance was accessed using classification accuracy (CA). It is a statistic that is frequently used to gauge how well machine learning algorithms are performing, defined by the number of correct predictions over the number of total predictions [43,46]. Precision, recall, and the F1 score were also computed. Recall is the proportion of correctly classified positive predictions among all true positive predictions; precision is the proportion of positively classified predictions that are true positives; and the F1 score is the harmonic mean of these two measures, and ranges from 0 to 1 [63]. A higher F1 score (closer to 1) indicates a better model. The performance metrics were calculated according to [64]. The associations of the alleles with the traits were also tested based on logistic regression and stepwise discriminant analysis at *p* < 0.05. Due to the considerably large training data for the mating type, metalaxyl response, and hyperparameter tuning, the overfitting problem for all the models was not a severe issue [65].

## 5. Results

**Figure 2:**
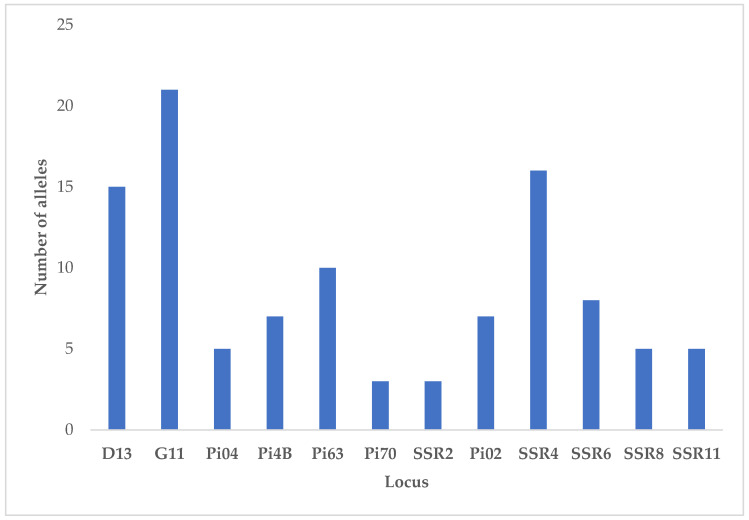
Locus and allele count of *P. infestans* isolates sampled for 12 loci from the Estonia, Pskov region, and Polish *P. infestans* population.

Among the *P. infestans* isolates characterized with SSR markers, 105 alleles were detected at 12 loci, all of which were polymorphic (Figure 2). They displayed allelic diversity among the isolates, with 3 (SSR2 and Pi70) to 21 alleles (G11) per locus, with an average of 8.75 ± 1.64 alleles. 

**Figure 3:**
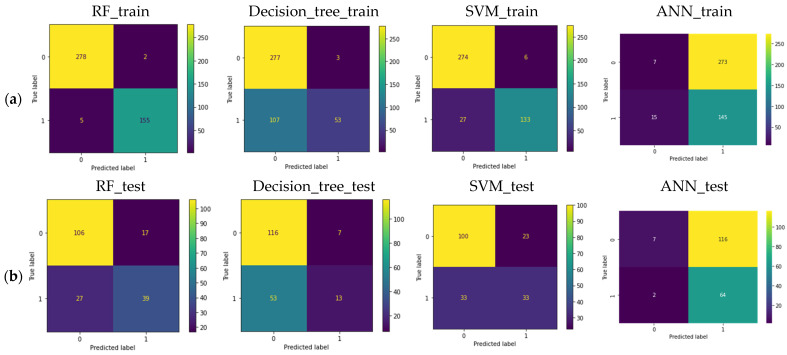
(**a**) Confusion matrix of training and (**b**) testing set for mating types (using all marker alleles) for random forest (RF), decision tree classifier, support vector machine (SVM), and artificial neural network (ANN). Coding as follows: 0—A1, 1—A2.

**Figure 4:**
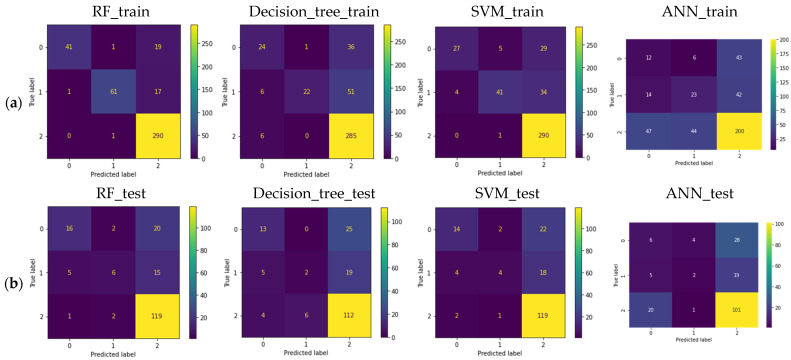
(**a**) Confusion matrix of training and (**b**) testing set for metalaxyl response (using all marker alleles) for random forest (RF), decision tree classifier, support vector machine (SVM), and artificial neural network (ANN). NB: Coded; 0: intermediate; 1: resistance; 2: sensitive.

The classification accuracy based on random forest, decision tree classifier, support vector machine, and artificial neural network for the mating type was 76.7%, 68.3%, 70.4%, and 37.6%, respectively, using all alleles (Figure 3b). For the metalaxyl response, classification accuracy based on random forest, decision tree classifier, support vector machine, and ANN for the metalaxyl response was 75.8%, 68.3, 73.7%, and 58.6%, respectively, using all alleles (Figure 4b). Using only common alleles, the classification accuracy based on random forest, decision tree classifier, support vector machine, and artificial neural network for the mating type was 75.7%, 67.2%, 67.7%, and 64.6%, respectively (Figure S1). For the metalaxyl response, classification accuracy using only common alleles based on random forest, decision tree classifier, support vector machine, and ANN for the metalaxyl response was 74.2%, 72.0%, 73.7%, and 63.4%, respectively (Figure S1). For all the machine learning models, random forest consistently gave a higher classification accuracy (typically above 75%) for both traits, followed by SVM (above 70%) using all alleles (Table 1). Likewise, using only common alleles, random forest consistently gave a higher classification accuracy (above 70%) for both traits (Table 1). This higher prediction for random forest is also reflected in the F1 score, which was consistently higher, compared to other models for both traits (Table 1). Compared to the other machine learning models, ANN ranked lower for classification accuracy for both traits when either all marker alleles or only common marker alleles were used. A total of 17 out of the 105 alleles were significantly (*p* < 0.05) associated with metalaxyl response (D13.136, D13.162, G11.160, G11.198, G11.164, G11.152, G11.204, Pi4B.205, Pi63.273, Pi63.151, SSR2.173, Pi02.258, Pi02.266, SSR4.292, SSR4.297, SSR6.232, SSR8.266). Only four alleles were significantly (*p* < 0.05) associated with mating types (G11.156, G11.202, G11.198, and Pi04.160).

## 6. Discussion

Machine learning has found applications in agriculture, including marker–trait association and genomic prediction [66,67,68]. Molecular marker technology has advanced our understanding in plant pathology research [69]. Among different classes of molecular markers, SSR markers have been widely used in numerous applications in plant pathology, such as genotyping pathogen populations, genetic diversity studies, monitoring changes in pathogen populations, phylogeny, parentage analyses, and reproductive biology of various plant pathogens [6,70,71,72,73]. This can be attributed to their multi-allelic nature, co-dominant inheritance (an important feature when analyzing the populations of diploid organisms such as *P*. *infestans*), relative abundance, repeatability, good genome coverage [74,75], and genotyping, which can be quickly completed by multiplexing all the pairs of SSR primers in a single reaction [76]. Indeed, in this study, all the loci were polymorphic, with most alleles found in the loci D13, SSR4, and G11, similar to those obtained by [77] in *P. infestans* genetic diversity studies using SSR markers.

In this study, different machine learning methods were applied in classifying mating type and metalaxyl fungicide response in *P. infestans* using SSR markers. The high classification accuracy across the models, except for the ANN that was considerably lower, indicates that these phenotypic traits likely have a genetic basis, and can be predicted using markers. Indeed, previous studies have linked DNA markers to metalaxyl response [26,35,36] and mating types [26,49], and several significant single nucleotide polymorphism markers (SNPs) have been associated with mating type and fungicide response in *Phytophthora* spp. [78]. The accuracy of the metalaxyl-sensitive response prediction was very high, ranging from 82.8% to 97.5%, compared to the prediction for other classes. The prediction of the A1 mating type was also more accurate compared to the prediction for the A2 mating type for all models except the ANN. This may be due to a larger training dataset for these classes. Ref. [36] observed that the allele 195 in locus Pi70 and the allele 166 in locus Pi04, which were common for all *P. infestans* populations in our study, were linked to metalaxyl-M (Mefenoxam) response. In our study, these alleles were not significantly associated with metalaxyl response. A total of 17 out of the 105 alleles were significantly (*p* < 0.05) associated with metalaxyl response. Comparably, several loci have been linked to insensitivity to metalaxyl in *P. infestans* [37,79]. However, only a few alleles were significantly associated with mating types, mainly in the G11 locus, indicating that mating types may be associated with a few major loci.

One would expect the ANN to perform better than other models due to the model architecture that closely mimics the human brain and its suitability for complex classification [80]. Yet, random forest was the best classifier for mating type and metalaxyl response. ANN models are more difficult to parametrize, and the choice of model parameters such as activation function, number of neurons, and number of hidden layers can greatly influence its performance. ANN has been shown to be accurate enough for the identification of pathogens and fungicide resistance and is thus suitable for use in practical situations [43]. Unlike the decision tree classifier, random forest as an ensemble method has greater accuracy, which can be attributed to its robustness in the selection of training samples and noise in the training dataset [81]. 

This study attempted to compare machine learning models for the classification of mating type and metalaxyl response in *P. infestans* using SSR markers. Although the classification accuracy can be deemed adequate in the machine learning context, particularly for the random forest model, it is debatable whether the accuracy is sufficient to guide decision-making. Moreover, in the plants within some machine learning studies, such as for the prediction of miRNAs associated with abiotic stresses [82], an accuracy of less than 70% was reported for some models. In humans, an accuracy of 65% to 70% was reported for diagnostic prediction of autism [83]. Even for genomic selection where markers are generally regarded as linked to phenotypes, an accuracy of less than 70% is common [84]. Thus, anything greater than 70% accuracy can be considered promising and has been reported, even with single nucleotide polymorphism markers [85,86,87,88]. However, our study shows a statistically significant association between the SSR marker alleles and metalaxyl response/mating type phenotypes, which opens up the possibility for the use of a slow-evolving SNP-based markers for the identification of both traits. On the other hand, incorporating more SSR markers and more datasets to balance the ratio of A1 and A2 mating types, as well as ratios of resistance and the intermediate and sensitive metalaxyl response in the training set, could also improve the quality of the dataset and thus the accuracy of models. Further hyperparameter optimization of the models can improve model performance and reduce any obvious overfitting. It can also be useful to examine the effect of decreasing or increasing the complexity of the models by feature engineering. Also, as high-throughput technology improves and as more markers are developed, a much higher predictive accuracy will likely be achieved. This kind of prediction could be a valuable addition to *P. infestans* population diversity studies based on SSR markers, aiding in the assessment of the metalaxyl fungicide effectiveness over time and space, and the possibility of sexual reproduction in the field (which can change the epidemiology of potato late blight and, in turn, changes the disease management strategies), especially when mating type and metalaxyl resistance data are not available. To ascertain the mating type and metalaxyl response, all that has to be done is input the unknown isolate’s SSR profile into a training model that has been developed using the SSR marker data training set. The program will then take up to a few seconds or minutes to execute its function. This technique may be used on both live and dead or archived biological samples, which makes it a useful addition to the toolkit for identifying mating type and metalaxyl response in *P. infestans* populations.

## Figures and Tables

**Table 1:** Overall prediction metrics for mating type and metalaxyl response based on all SSR markers alleles and common SSR markers alleles.

**All SSR Alleles**	**Mating Type**	**Accuracy**	**Precision**	**Recall**	**F1_Score**
	Random Forest	76.72	0.726	0.747	0.736
	Decision Tree	68.25	0.570	0.668	0.615
	Support Vector Machine	70.37	0.657	0.671	0.663
	Artificial Neural Network	37.57	0.513	0.567	0.539
	**Metalaxyl response**	**Accuracy**	**Precision**	**Recall**	**F1_Score**
	Random Forest	75.81	0.542	0.700	0.611
	Decision Tree	68.28	0.446	0.520	0.480
	Support Vector Machine	73.66	0.499	0.673	0.573
	Artificial Neural Network	58.60	0.354	0.387	0.370
	**Mating type**	**Accuracy**	**Precision**	**Recall**	**F1_Score**
**Common SSR Alleles**	Random Forest	75.66	0.718	0.733	0.726
	Decision Tree	67.20	0.565	0.634	0.598
	Support Vector Machine	67.72	0.619	0.637	0.628
	Artificial Neural Network	64.55	0.559	0.585	0.572
	**Metalaxyl response**	**Accuracy**	**Precision**	**Recall**	**F1_Score**
	Random Forest	74.19	0.538	0.652	0.590
	Decision Tree	72.04	0.473	0.670	0.555
	Support Vector Machine	73.66	0.509	0.691	0.586
	Artificial Neural Network	63.44	0.453	0.489	0.471

## Data Availability

The original contributions presented in the study are included in the article and Supplementary Material, further inquiries can be directed to the corresponding author.

## Supplementary Materials

The following supporting information can be downloaded at: https://www.mdpi.com/article/10.3390/microorganisms12050982/s1. Figure S1: Metalaxyl response and mating type accuracy for the different machine learning models using common SSR marker alleles; Table S1: Changes in allele frequency of SSR marker alleles of *P. infestans* isolates, for common and all alleles across populations for mating type and metalaxyl response.
