# Supplementary material for: Machine Learning-Based Identification of Mating Type and Metalaxyl Response in Phytophthora infestans Using SSR Markers
Source: Microorganisms. 2024 May 14;12(5):982. doi: 10.3390/microorganisms12050982 (PMC11124124; doi:10.3390/microorganisms12050982)

Figure S1. Metalaxyl response and mating type accuracy for the different machine learning models using common SSR marker alleles

Metalaxyl Response: Random Forest

Prediction accuracy.
(a). Training Set: 98.61 %

(b). Test Set: 74.19 %

a

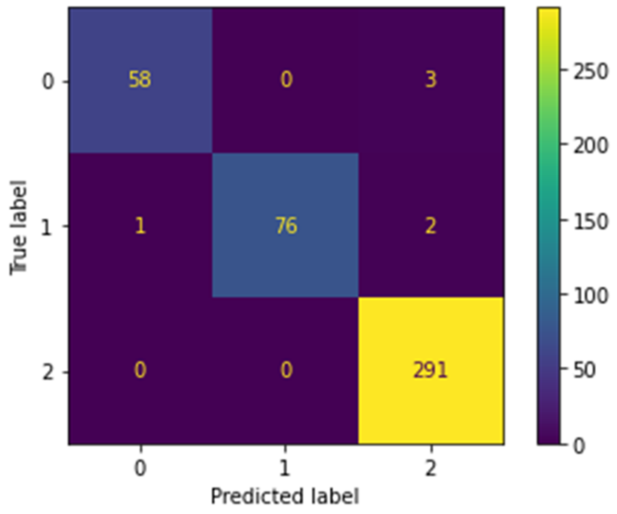

b

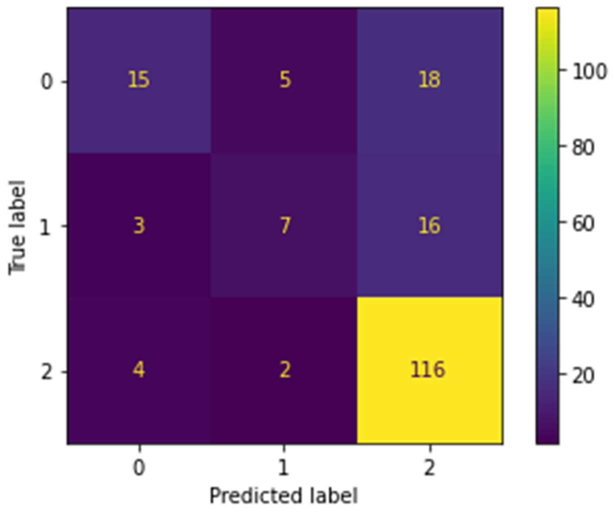

Metalaxyl Response: Decision tree

Prediction accuracy.

(a). Training Set: 75.17 %

(b). Test Set: 72.04 %

a

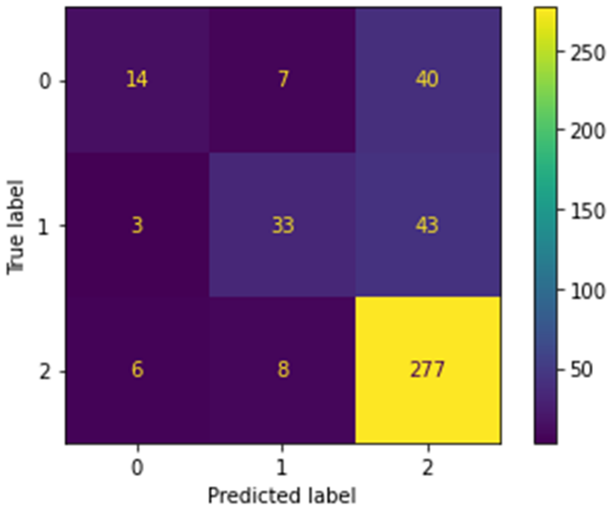

b

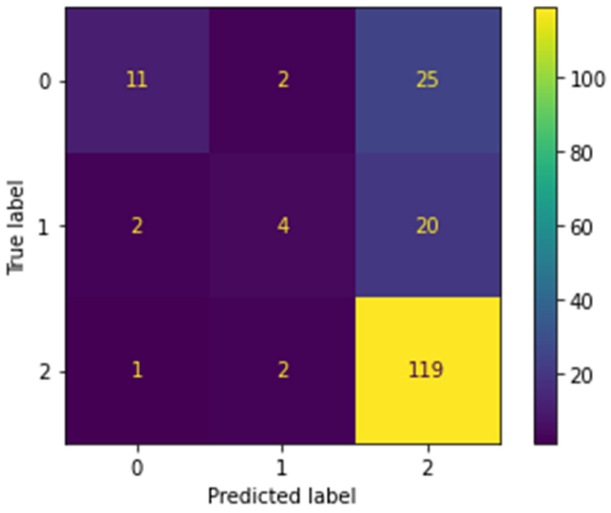

Metalaxyl Response: Support vector machine

Prediction accuracy.
(a). Training Set: 83.29 %

(b). Test Set: 73.66 %

a

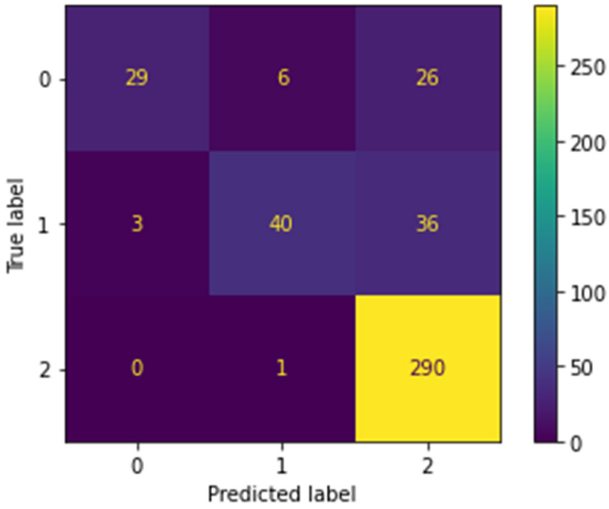

b

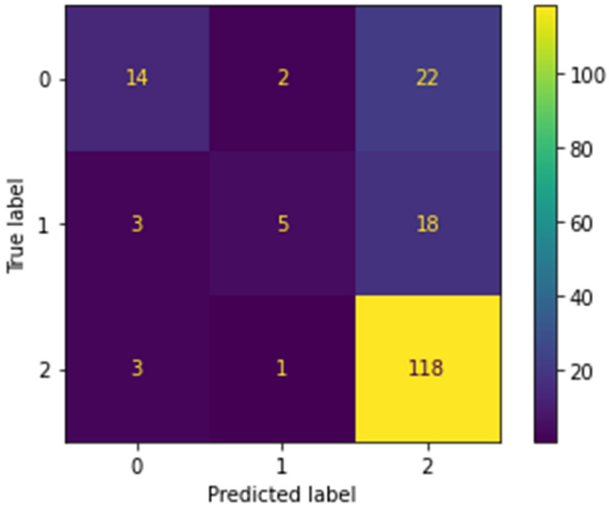

Metalaxyl Response: Artificial neural network

Prediction accuracy.
(a). Training Set: 75.87%
(b). Test Set: 63.44%

a

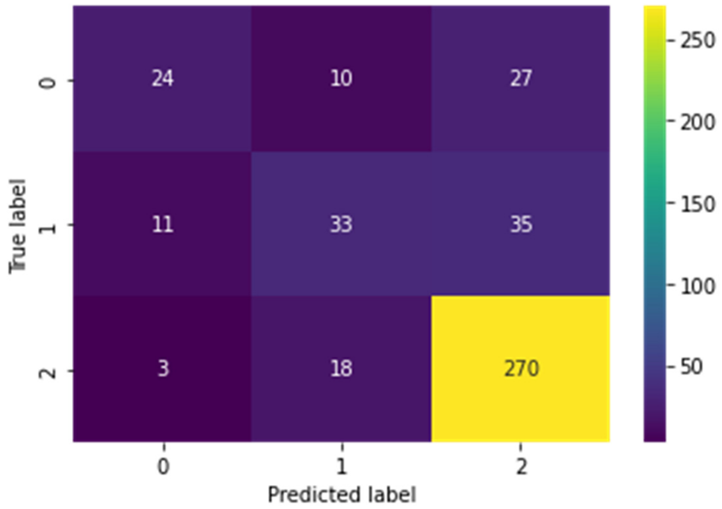

b

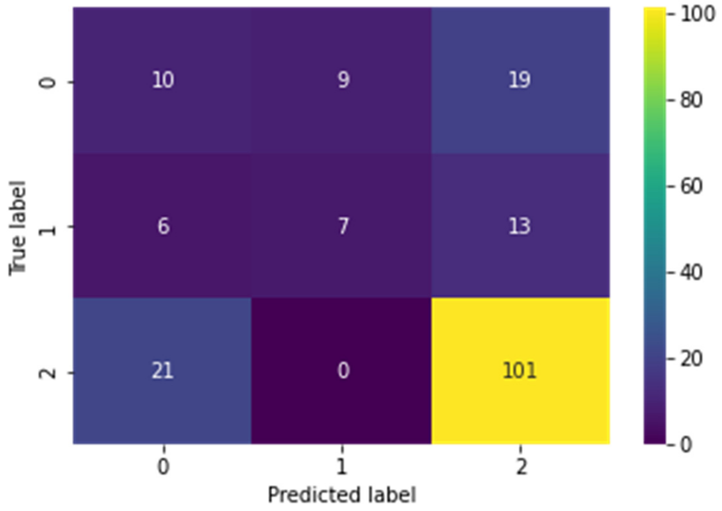

Mating type: Random Forest

Prediction accuracy.

(a). Training Set: 98.41 %

(b). Test Set: 75.66 %

a

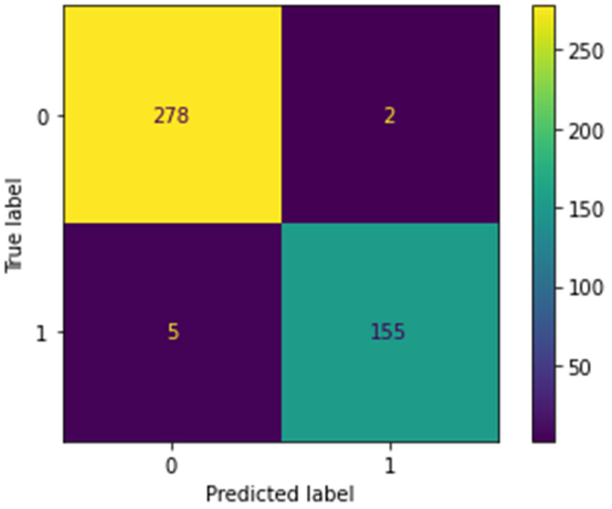

b

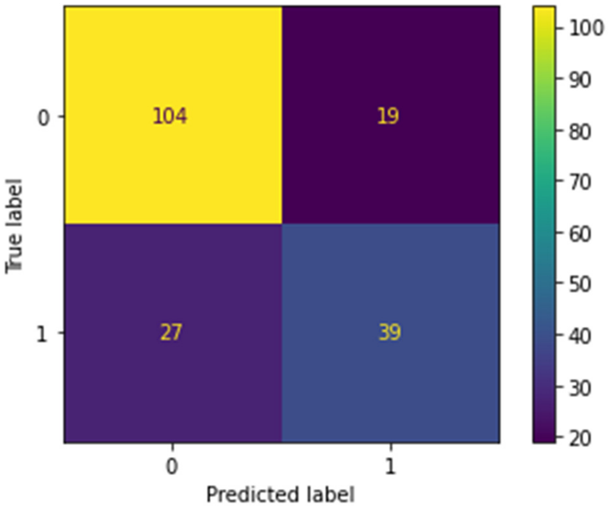

Mating type: Decision tree

Prediction accuracy.

(a). Training Set: 76.59 %

(b). Test Set: 67.2 %

a

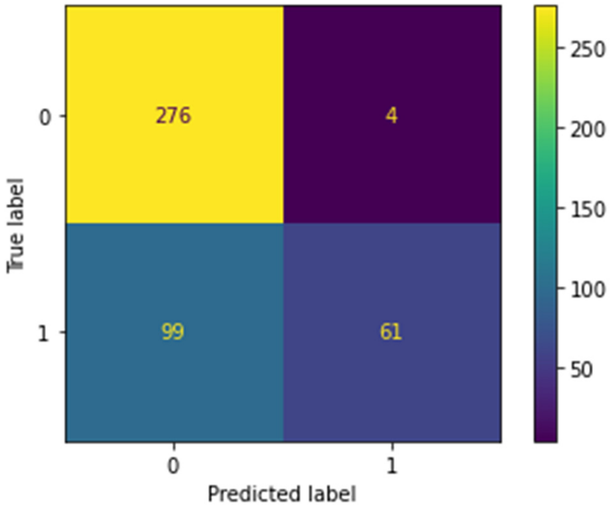

b

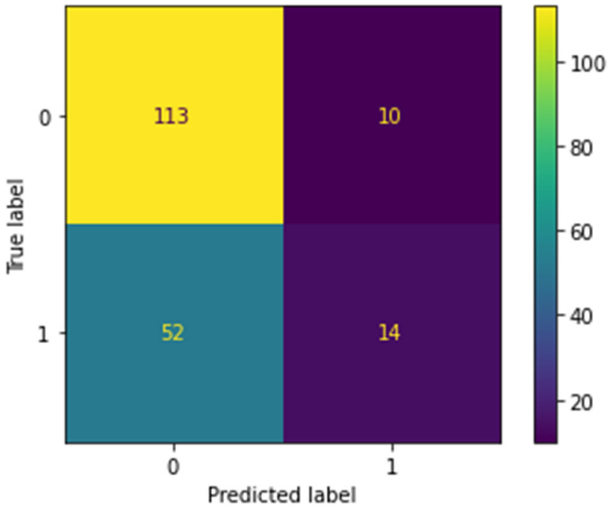

Mating type: Support vector machine

Prediction accuracy.
(a). Training Set: 89.09 %

(b). Test Set: 67.72 %

a

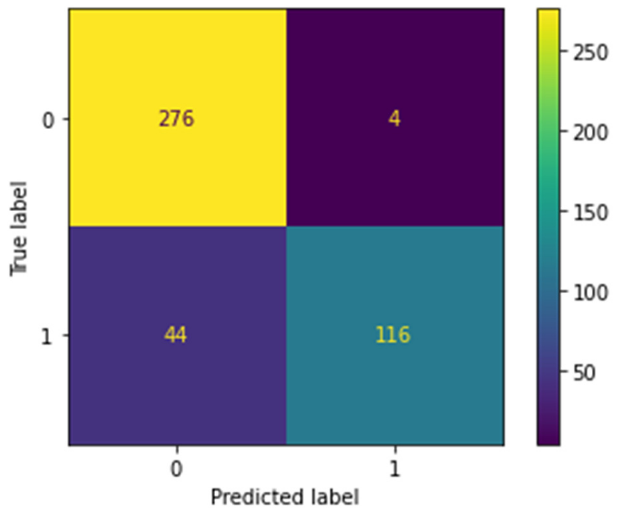

b

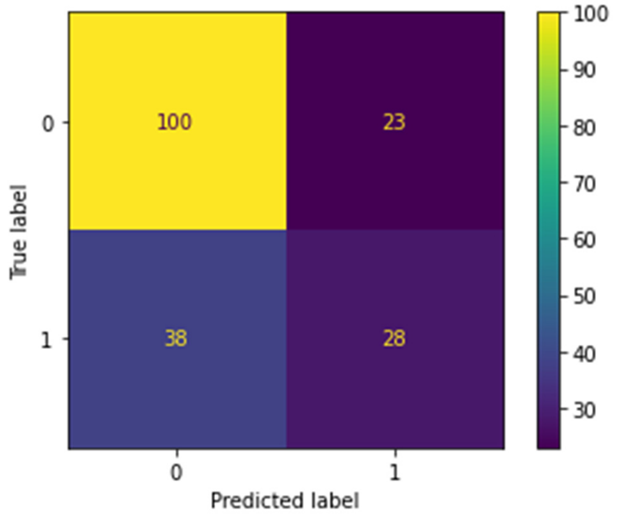

Mating type: Artificial neural network

Prediction accuracy.
(a). Training Set: 73.41 %

(b). Test Set: 64.55 %

a

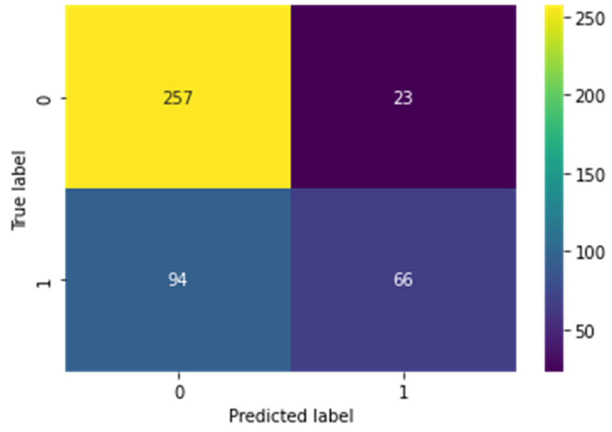

b

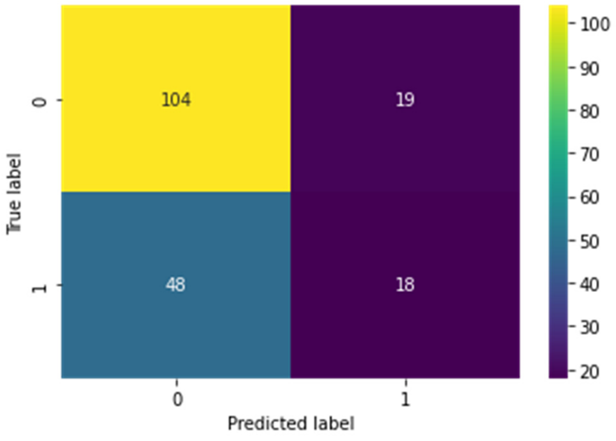

Supplement: Supplementary file 1 [file microorganisms-12-00982-s001.zip › Figure S1.pdf]
